# A computational model of cerebrospinal fluid production and reabsorption driven by Starling forces

**DOI:** 10.3325/cmj.2014.55.481

**Published:** 2014-10

**Authors:** Joel Buishas, Ian G. Gould, Andreas A. Linninger

**Affiliations:** UIC Department of Bioengineering, University of Illinois at Chicago, Chicago, IL, USA

## Abstract

Experimental evidence has cast doubt on the classical model of river-like cerebrospinal fluid (CSF) flow from the choroid plexus to the arachnoid granulations. We propose a novel model of water transport through the parenchyma from the microcirculation as driven by Starling forces. This model investigates the effect of osmotic pressure on water transport between the cerebral vasculature, the extracellular space (ECS), the perivascular space (PVS), and the CSF. A rigorous literature search was conducted focusing on experiments which alter the osmolarity of blood or ventricles and measure the rate of CSF production. Investigations into the effect of osmotic pressure on the volume of ventricles and the flux of ions in the blood, choroid plexus epithelium, and CSF are reviewed. Increasing the osmolarity of the serum via a bolus injection completely inhibits nascent fluid flow production in the ventricles. A continuous injection of a hyperosmolar solution into the ventricles can increase the volume of the ventricle by up to 125%. CSF production is altered by 0.231 µL per mOsm in the ventricle and by 0.835 µL per mOsm in the serum. Water flux from the ECS to the CSF is identified as a key feature of intracranial dynamics. A complete mathematical model with all equations and scenarios is fully described, as well as a guide to constructing a computational model of intracranial water balance dynamics. The model proposed in this article predicts the effects the osmolarity of ECS, blood, and CSF on water flux in the brain, establishing a link between osmotic imbalances and pathological conditions such as hydrocephalus and edema.

Cerebrospinal fluid (CSF) is contained in the ventricles of the brain as well as the cranial and spinal subarachnoid spaces (SAS). The total volume of CSF is 150 mL for the average adult where 125 mL is confined to the subarachnoid spaces and 25 mL to the ventricles (1). CSF is renewed four or five times over a 24 hour period (1) and the total volume of CSF produced is roughly 580 mL per day. The composition of the CSF and plasma are superficially different. CSF has been shown to contain higher concentrations of Na^+^, Cl^-^, Mg^2+^, while the concentrations of glucose, albumin, K^+^, HCO_3_^-^, Ca^2+^, and phosphate are lower than in the serum (2-4). The main functions of CSF are to protect the brain from mechanical injury, flush the brain of metabolites, and act as a water reservoir for the parenchyma.

There is a fundamental lack of understanding of the basic mechanisms that drive production, reabsorption, and flow of CSF within the brain. The classical hypothesis of active CSF secretion by the choroid plexus and absorption via the arachnoid granulations does not explain the experimental failure of surgical inhibition to relieve the symptoms of hydrocephalus. Traditional in vivo methods of studying CSF physiology have serious deficiencies that cast doubt on the classical view of CSF flowing as a river from the choroid plexus to the arachnoid villi. Additionally, anatomical discrepancies between animal and human models make it difficult to extrapolate the mechanism of production and absorption. All these factors complicate development of a physiologically accurate model of the production, reabsorption, and exchange of CSF.

## The classical model of CSF production by the choroid plexus

**Figure 1:**
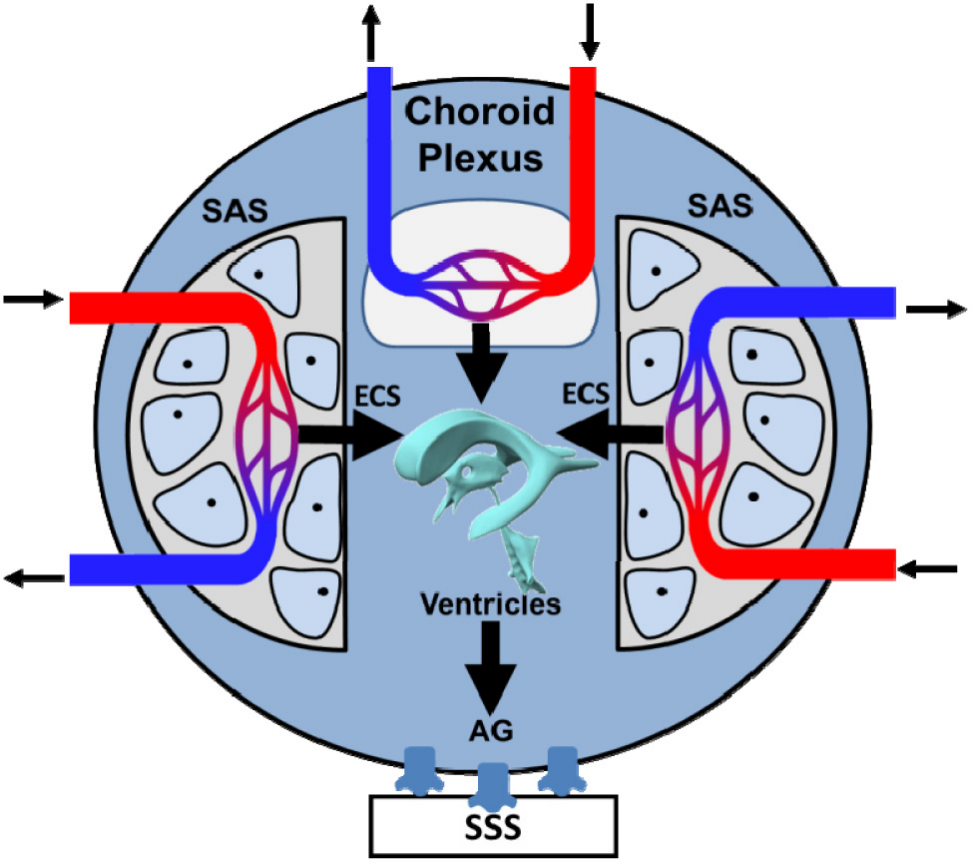
Simplified illustration depicting both the classical and the microvessel hypothesis. The choroid plexus actively creates an ionic concentration gradient between the blood and the ventricles, which drives water transport. The cerebrospinal fluid (CSF) then flows through the ventricles, where it is absorbed through the arachnoid granulations (AG) into the superior sagittal sinus (SSS). CSF is produced and reabsorbed by the capillaries in the parenchyma due to an imbalance in hydrostatic and osmotic pressure, known as Starling force.

The classical hypothesis postulates that the choroid plexus epithelium, located in the ventricles, actively secretes CSF. The choroid plexus is composed of specialized epithelial cells that contain mitochondria, which are joined together via tight junctions. Membrane-bound transporters of Na^+^, K^+^, Cl^-^, and HCO_3_^-^ ions on both the apical (CSF facing) and basolateral (blood facing) surface of the choroid plexus epithelium create a concentration gradient. This gradient drives water filtration from the fenestrated arterials feeding the choroid plexus into the CSF in the ventricles (5,6). In the classical view, newly formed CSF flows to the subarachnoid space where it passes through the arachnoid granulations into the superior sagittal sinus (SSS). The production of CSF and movement through the ventricles to the veins is illustrated in Figure 1.

There is a significant body of work that suggests the choroid plexus is an integral element of CSF production (7-16). A choroid plexus is located in each lateral ventricle, the third, and the fourth ventricle. Together, these structures represent 200 cm^2^ of secretory area (17-19) with a combined weight (9) of 2 g. The hypothesis that the choroid plexus actively secretes CSF is derived from the fenestrations of periventricular arterioles, the magnitude of blood flow to the choroid plexus, and the morphology of the choroid plexus epithelium. The blood flow to the choroid plexuses is estimated (10) to be 3 mL/min/g: a full order of magnitude higher than the flow to the brain parenchyma (20) and five times higher than the blood flow to other secretory organs like the kidney (21). Additionally, periventricular capillaries that feed the choroid plexus are fenestrated, unlike the capillaries of the surrounding parenchyma whose capillaries are joined by tight junctions (22). The choroid plexus contains both apical microvilli and basolateral folding to increase the surface area contacting both the blood and CSF (9). The blood brain barrier in the choroid plexus is leaky as shown by horse radish perioxidase (HRP) studies (23). In the parenchyma, HRP does not leave the cortical microcirculation.

## Evidence against the choroid plexus as the main source of CSF

Experimental evidence casts doubt on the classical hypothesis that the choroid plexus is the main producer of CSF (7,24-31). The CSF production of 580 mL/d is unlikely to be explained by surface area of the choroid plexus (200 cm^2^) alone when compared to the cerebral capillaries (140 000 cm^2^). *In vitro* experiments (7) utilizing cell cultures of porcine choroid plexus epithelial cells have demonstrated that the cells were able to produce 48.2 µL/cm^2^ per hour, which corresponds to only 243 mL/d. Additionally, the classical hypothesis cannot explain the failure of surgical inhibition of the choroid plexus to relieve the symptoms of hydrocephalus. Surgical inhibition is accomplished via the insertion of an endoscope and a unipolar electrode wire into the occipital horn of the lateral ventricle. The electrode is then used to coagulate the choroid plexus until it exhibits a white color and shriveled morphology (24). In a study by Pople and Ettles (24), coagulation of the choroid plexus did not significantly reduce the size of the ventricle and 65% of patients required a CSF shunt to achieve long term control of symptoms. These studies suggest that there exist additional sources of CSF production that enables water and solute transport between cerebral capillaries, the extracellular space (ECS), and the ventricles.

## The classical model of CSF reabsorption through the arachnoid granulations

The classical hypothesis of CSF absorption postulates that CSF flows as a river from the site of production in the choroid plexus in the ventricles to the arachnoid granulations (AG) into the SSS, the large vein that drains the cerebral vasculature. These AG are located at the interface between the subarachnoid space and the SSS (32). Endothelial cell-lined channels within these granulations (33) transport CSF from the subarachnoid space into the veins. In experiments using cultured human arachnoid cells, significant increase in flow rates and cellular hydraulic conductivity was observed only when the membrane was perfused in the physiological direction of flow (34). These results suggest that the AG act as one-way valves. The communication between the CSF and veins through the AG has been investigated using an extracted section of simian dura matter for a size exclusion study (35). Smaller particles passed through, while larger particles became enmeshed in the villi, thus impeding the flow. This experiment demonstrates a pathway from the CSF to the veins through the AG for water and solutes. It is thought that 85%-90% of CSF reabsorption occurs at intracranial sites, while 10%-15% is absorbed via spinal sites (2,4), therefore, spinal absorption will be ignored in this model. This empirical evidence shows that the absorption of CSF through AG needs to be considered in order to create a physiologically accurate computational model.

## The perivascular space

Recent experimental evidence suggests that there exists a pathway for the movement of water and solute in the perivascular space (PVS) surrounding the blood vessels in the brain (36-38). This space is the annular region between the epithelium lining the vessel and either the pial sheath or the glial limitans (39-45). It has been shown by Iliff et al (36-38) that radioactive tracers injected into the SAS are rapidly dispersed into the interstitial space by penetrating arteries. The speed at which radioactive tracer was distributed throughout the brain parenchyma suggests that there is convective flow of water and solute in the perivascular space that connects the CSF to the parenchyma. A complete phenomenological model of water and solute transport in the brain should include the perivascular space.

## The microvessel model of CSF production and reabsorption

Studies linking the production and reabsorption of CSF with the osmolarity of the blood and ventricles (25-30) suggest that the normal balance of osmolites in the blood, ECS, and ventricles plays a role in the balance of water between these brain compartments. The Monro-Kellie doctrine states that the sum of volumes of brain, CSF, and intracranial blood must remain constant. *In vivo* measurements are obtained using ventriculo-cisternal perfusion (VCP) to measure the effect of osmolarity on the bulk flow of nascent fluid, which constitutes CSF production by the choroid plexus and any bulk that is drawn into the ventricle as the result of an osmotic gradient.

Radiolabeled water experiments were also conducted that utilized an injection of ^3^H_2_0 into either the lateral ventricle or the bloodstream and compared the radioactivity of the blood and the CSF in the cisterna magna.

These experiments suggest that water is not confined to CSF space or blood lumen, but is constantly interchanged between the parenchyma and the blood throughout the brain. These observations form the basis of the microvessel hypothesis in which water transport between the blood, ECS, and ventricles is driven by Starling forces. The microvessel hypothesis (46-49) has been proposed in response to the inability of the classical hypothesis to explain empirical evidence. It states that the microvessels in the central nervous system contribute to CSF production and reabsorption based on the relationship between hydrostatic and osmotic pressure gradients created by both proteins and inorganic ions across the capillary membrane. This relationship is described by the osmotic counter pressure hypothesis (47-49), which proposes that plasma osmolytes are retained as water is filtered out of capillaries due to hydrostatic pressure, causing osmolytes to concentrate and drive the reabsorption of water into post capillary venules or veins. Osmotic pressure gradients have been found to be a significant driving force in the transport of water (25-30) and solute (30) between the blood, parenchyma, and the ventricles. The interaction of colloid osmotic pressure and hydrostatic pressure on transmembrane water transport is described by the Starling oncotic hypothesis of capillaries. However, this hypothesis is insufficient to describe mass transport phenomena in the central nervous system (47) because of the tight junctions joining the endothelial cells in the cerebral capillaries. These tight junctions create zona occludens (17,50), which prevents the diffusion of ions across the capillary wall in the cerebral microcirculation, which contributes to the osmotic pressure (17,47,50,51). An amended Starling’s law (17,47) is implemented to include the contribution of proteins, ions, and glucose to the total osmotic pressure across a membrane. The term Starling forces is also expanded (17) to include both colloid osmotic pressure and osmotic pressure generated from ions and glucose.

**Figure 2:**
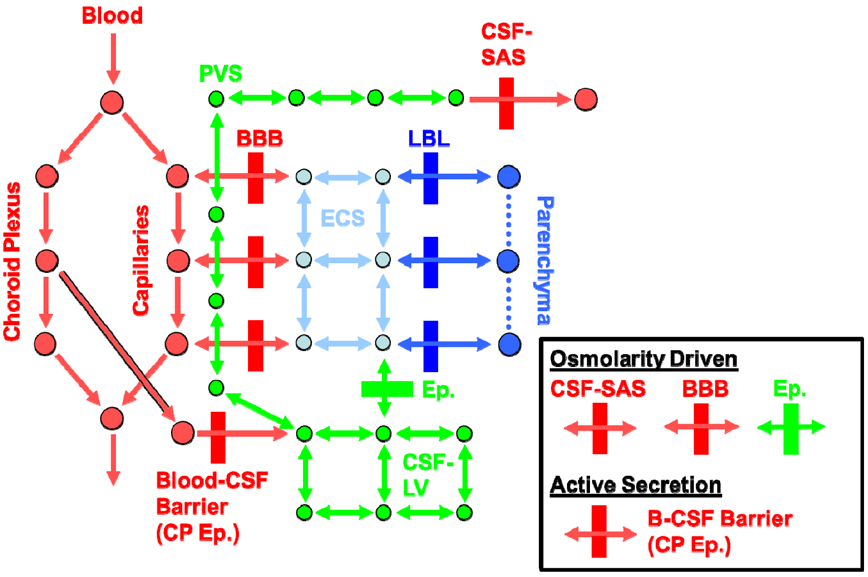
Pathways for water exchange in the brain. In addition to blood flow through the vasculature, water can also be filtered through the epithelium of the blood vessels and pass through the blood-brain barrier (BBB) to communicate with the extracellular space (ECS) or the perivascular space (PVS) driven by Starling forces, where it would either flow into the ventricles or leave the system through the subarachnoid space (SAS). Once in the ECS, water can pass between cellular membranes (CM) in the parenchyma or between the Ependymal layer (Ep.) of the ventricles (CSF-LV) following osmotic gradients, where it would flow through the ventricular system and into the SAS before being drained into the superior sagittal sinus. Water can also be transported from the blood to the ventricles via filtration through the choroid plexus epithelium (CP Ep.), which forms the blood-CSF barrier by an active process.

Figure 2 depicts a schematic for the possible pathways for water exchange in the brain. Blood flows through the vasculature from arteries to capillaries to veins. At the capillary level most of the water flows through the vasculature. A small fraction is believed to filter through the blood brain barrier to communicate with the ECS, or into the perivascular spaces. The perivascular space allows water to flow to either the ventricles or the SAS. In the ECS, water is transported back into the downstream capillaries across the blood-brain barrier, through the cellular membrane of cells in the parenchyma, or across the ependymal layer of the ventricles into the CSF. Water can be transported from the capillaries that feed the choroid plexus through the blood-CSF barrier into the ventricles.

Ample experimental evidence suggests that the transport of water between the blood, ECS, and ventricles is driven by osmotic pressure gradients (25,27-30,52,53).

The goal of this investigation is to present a phenomenological description of CSF production and reabsorption, describe the experimental methods used to study the physiology of CSF, and gather relevant data from those experiments. These observations were then compiled into a novel computational model that can be used to interrogate the effects of osmolarity gradients on water accumulation in the ECS and CSF. The methods section of this article describes experimental techniques used to study the physiology of CSF formation and the effect of osmolarity on the flow rate of CSF and ventricular volume. The results section presents empirical evidence for the active transport of ions^-^ across the choroid plexus epithelium. Additionally, the effect of osmolarity on the rate of CSF production and water transport within the brain is described. The discussion section introduces a novel computational model that synthesizes the classical hypothesis with the microvessel hypothesis. The conclusion outlines how the computational model can be used to examine the effect of osmolarity on intracranial pathology including hydrocephalus and edema.

## Methods

The following section summarizes the methods used to study CSF physiology. Protocols for measuring the effect of osmolarity of the serum and ventricle on bulk flow rates of nascent fluid and ventricular volume are described. The measurement of ionic concentrations in the blood, CSF, and choroid plexus epithelium is also explained. Finally, methods for determining the flux of ions across the choroid plexus epithelium are presented.

### Ventriculo-cisternal perfusion method

**Figure 3:**
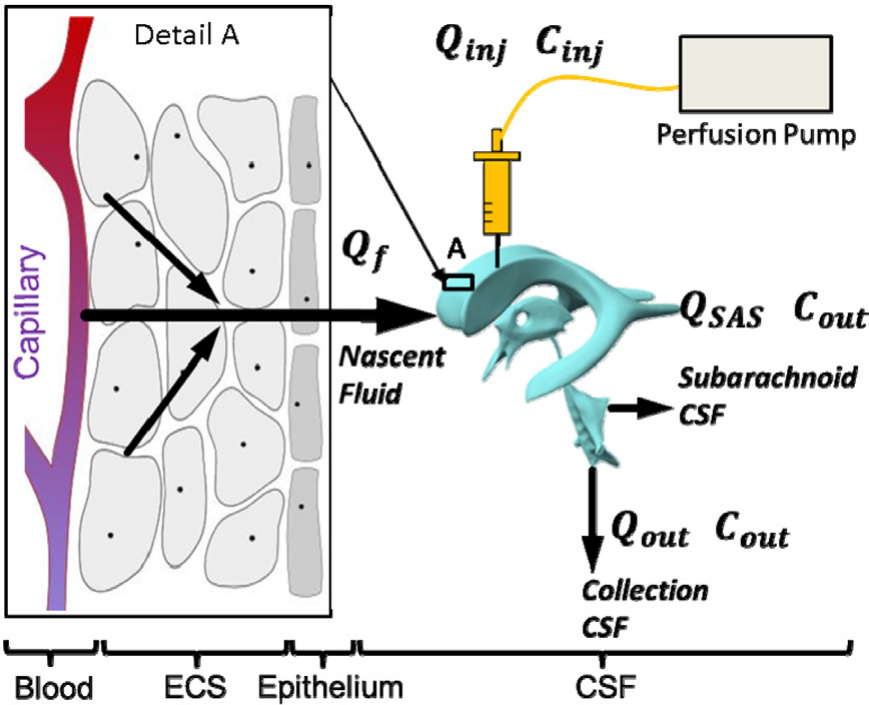
Ventriculo-cisternal perfusion method for studying the physiology of cerebrospinal fluid (CSF) production. The lateral ventricle is continuously injected with artificial CSF containing a dye. Nascent fluid is defined as any fluid that is drawn into the ventricle as a result of osmotic pressure from the capillary bed throughout the extracellular space (ECS) and epithelium. The flow rate of nascent fluid into the ventricle is determined based on the dilution of the dye measured in the collection fluid from the cisterna magna. Q_f_ is calculated using equation 1-2 where Q_inj_ is the bulk flow rate of the perfusion fluid, Q_out_ is the bulk flow rate of the collection fluid, Q_SAS_ is the bulk flow rate of the fluid in the SAS, C_inj_ is the tracer concentration in the perfusion fluid, and C_out_ is the tracer concentration in the collection fluid.




Ventriculo-cisternal perfusion (VCP) measures the effect of osmolarity on the formation of CSF (Figure 3). An infusion pump injects a perfusion solution containing a tracer via an inflow cannula into the lateral ventricle. The osmolarity of the ventricle is altered by injecting an anisotonic solution into the ventricle, either via a continuous or bolus injection. Fluid is then collected from the outflow cannula inserted into the cisterna magna. This procedure allows for indirect measurement of the production rate of nascent fluid, the volumetric flux of water into the lateral ventricle from either the choroid plexus or through the ependymal layer from the ECS. The rate of nascent fluid formation is calculated using equation 1-2 where Q_inj_ is the bulk flow rate of the perfusion fluid, Q_out_ is the bulk flow rate of the collection fluid, Q_SAS_ is the bulk flow rate of the fluid in the SAS, C_inj_ is the tracer concentration in the perfusion fluid, and C_out_ is the tracer concentration in the collection fluid.

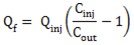
[1]


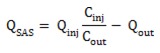
[2]

The values of Q_SAS_ and Q_f_ are unknown. The effect of osmolarity on the bulk flow of nascent fluid in the ventricles is examined by altering the osmolarity of either the blood (26,29,30) or the lateral ventricle (25,27,28).

### VCP experiments measuring the effect of osmolarity in bulk flow of nascent fluid

This section summarizes the body of literature on osmolarity induced changes in CSF production. The effect of altering the osmolarity of the serum and the ventricle on the bulk flow of nascent fluid in animals undergoing VCP was examined in the following studies. The experiments are grouped by their protocol. Experiments (E) 1 and 2 altered the osmolarity of the ventricles, while experiments 3-5 altered the osmolarity of the serum. The data collected in these experiments are described in the Results section.

*E1. Ventricular osmolarity and the bulk flow of nascent fluid.* Wald et al (25) measured the effect of changes in the osmolarity (osmotic loading) of the ventricles on the CSF production during VCP by indirectly measuring the bulk flow of nascent fluid. The non-diffusible tracer ^131^I-labeled cat serum albumin was used to determine the bulk flow rate of nascent fluid. Ventricles were injected with mock CSF at a rate of 77 µL/min with an osmolarity of 320 mOsm. Each cat served as its own control. The cats were then injected with a sucrose solution at the same rate with an osmolarity of between 6-780 mOsm. Samples of collection fluid from the cisterna magna were taken in 15-minute intervals. Perfusion was maintained until steady state, defined as when the indicator concentration in the collection fluid for successive samples differed by less than ±2%.

*E2.Ventricular osmolarity and the fate of radiolabeled water.* Klarica et al (27) traced the transport of radiolabeled ^3^H_2_0 from the blood into the cisterna magna and measured the effect of injection of hyperosmolar solution into the lateral ventricle of the canine model by measuring the radioactivity of the collection fluid from the cisterna magna. Radiolabeled water was injected into the bloodstream and its dilution was measured in response to a hyperosmolar bolus injection of a 950 mM sucrose solution into the lateral ventricle. Blood samples were collected from the femoral artery and injections of ^3^H_2_O were made in the femoral vein. ^3^H_2_O concentrations were measured in the lateral ventricle by VCP as described in the previous section. Equilibrium was reached when the concentration of ^3^H_2_O in the collection fluid and the arterial plasma measured by a liquid scintillation counting were equal.

*E3. Serum osmolarity and bulk flow of nascent fluid.* The effect of serum osmolarity on the bulk flow of nascent fluid into the ventricles in a feline model was examined by Dimattio et al (26). Glucose solutions ranging from 290-360 mOsm were infused at a rate of 2.2 mL/min for a total of 15 minutes into the femoral vein to alter the serum osmolarity during VCP. The cats were continuously perfused with artificial CSF with an osmolarity of 320 mOsm. Perfusion of ^125^I-labeled cat serum albumin was injected at a rate of 77 µL/min and collection fluid was sampled at 15 minute intervals.

*E4. Serum osmolarity and the rate of solute transport from blood to CSF.* The effect of decreasing the serum osmolarity on the rate of CSF production and the movement of solute from the cortical white matter into the cisterna magna was examined by Wald et al (30) by measuring the radioactivity of the collection fluid in the cisterna magna. During VCP a solution of 20 µL saline, 40 mg/mL dextran blue, and 0.5-1 µmoles of ^22^NaCl was injected over 5 minutes into the cerebral cortical white matter. Mock CSF with an osmolarity of 320 mOsm was perfused at a rate of 77 µL/min. The radioactivity of the collection fluid from the cisterna magna was examined before, during, and after an induced decrease in blood serum osmolarity. Tonicity of the blood was adjusted by intravenously administered injection of hypo-osmolar 60 mOsm sucrose solution at a rate of 2.2 mL/min.

*E5. Increasing serum osmolarity and the bulk flow of nascent fluid.* Increasing serum osmolarity with a bolus injection of hyperosmolar sucrose was measured by Jurjevic et al (29). CSF outflow rates in cats were measured before, during, and after the IV administration of a 1110 mOsm mannitol solution during VCP with a perfusion of mock CSF at 12.96 mL/min. The injection of a hyperosmolar solution was made into the femoral vein. Measurements of nascent fluid flow were taken every 30 minutes (Table 1).

**Table 1:** Summary of ventriculo-cisternal perfusion experiments

Experiment	Author (Year)	Injection	Injection location	Measurement
E1	Wald (1976) (11)	Continuous	Lateral ventricle	Nascent fluid flow rate
E2	Klarica (2013) (13)	Bolus	Lateral ventricle	Collection radioactivity
E3	Dimattio (1975) (12)	Continuous	Femoral vein	Nascent fluid flow rate
E4	Wald (1977) (16)	Bolus	Femoral vein	Collection radioactivity
E5	Jurjevic (2012) (15)	Bolus	Femoral vein	Nascent fluid flow rate

### Non-VCP experiments examining CSF physiology

The following experiments examine water transport between the compartments of the brain by investigating ventricular volume changes as well as ionic concentration gradients. The effect of continuous injection of hyperosmolar solution into the lateral ventricle on the volume of the ventricle was measured, as well as the concentration of ions in the blood, CSF, and the choroid plexus epithelium. The flux of key ions between the blood and CSF was measured as well.

*E6. Effect of osmotic loading of the ventricle on ventricular volume.* The effect of a continuous injection of hyperosmolar solution into the lateral ventricle on ventricular volume was measured by two different medical imaging methods. The first method utilized planemetry (27), where coronal slices of the cat brain were obtained 7 days after continuous injection of a hyperosmotic solution. The contours of the coronal slice and the lateral ventricles were projected onto a screen and traced with millimeter paper. The second method measured ventricular enlargement with magnetic resonance imaging (MRI), as the percentage cross-sectional area of the ventricle over the total brain surface of three coronal slices. By manually outlining the region of interest and using a computer to count the pixels, the volume change was given by the percent increase when compared to the control (53).

*E7. Measuring ionic concentration in the blood, CSF, and choroid plexus (E7a, E7b).* According to classical hypothesis of CSF physiology, the active transport of Na^+^, K^+^, Cl^-^, and HCO_3_^-^ between the fenestrated arterioles feeding the choroid plexus, the choroid plexus epithelium, and the CSF in the ventricles creates an osmotic gradient that drives CSF production. The Na^+^ and K^+^ concentration in the blood, CSF and choroid plexus epithelium were determined via flame atomic emission spectrometry (11-16), where the sample was exposed to a non-luminous flame, and photoelectric circuitry determined the ionic contents of the sample (54). Concentrations of Cl^-^ were measured by amperometric titration of silver in a chloridometer. Bicarbonate concentration was determined indirectly by first measuring arterial pH and pCO_2_ of a sample with micro electrodes and the Henderson-Hasselbalch equation (55) as shown in equation 3. The p*K*_a_ is the log acidity constant of carbonic acid (6.1), and Kh is the Henry's law constant for the solubility of carbon dioxide in blood (0.03 mmol/mL/mmHg).


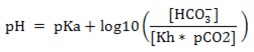
[3]

The ionic concentration of Na^+^, K^+^, Cl^-^, and HCO_3_^-^ in the choroid plexus epithelium was determined by a method devised by Johanson et al (13-16). The choroid plexuses in the fourth and lateral ventricles were surgically removed with a stereoscopic dissecting microscope and ophthalmologic forceps. The tissue was then dried and analyzed for ionic concentrations in the manner described previously. Blood samples were taken from the femoral artery (11,12) or the aorta (13,14). CSF samples are either taken from the cisterna magna (22-25) or newly formed fluid directly from the choroid plexus epithelium (11,12). In this method, cats were anesthetized and artificially respirated before the removal of one side of the skull and roof of the lateral ventricle to expose the choroid plexus, which was then covered with Pantopaque oil. The fluid that formed in between the epithelium and the oil was collected utilizing a micropipette technique (11,12).

*E8. Determining the ionic flux across the choroid plexus epithelium.* The fluxes of Na^+^, K^+^, and Cl^-^ across the choroid epithelium were determined utilizing a method developed by Wright et al (8), where the choroid plexus from the fourth ventricle of the bull frog was surgically removed and placed in an Ussing chamber, which consists of two Lucite half chambers that secure the tissue in place. The flux measurements across the choroid epithelium were taken by adding radioactive isotopes in a solution to one side of the chamber and sampling 100 µL of fluid every 20 min. The radioactive samples were then assayed using a gas flow proportional counter.

## Results

The following section includes a summary of the empirical results from the experiments described in the Methods section. The effects of both ventricle and serum osmolarity on CSF production are described, as well as the measurement of ionic concentrations in the blood, CSF, and choroid plexus epithelium.

**Figure 4:**
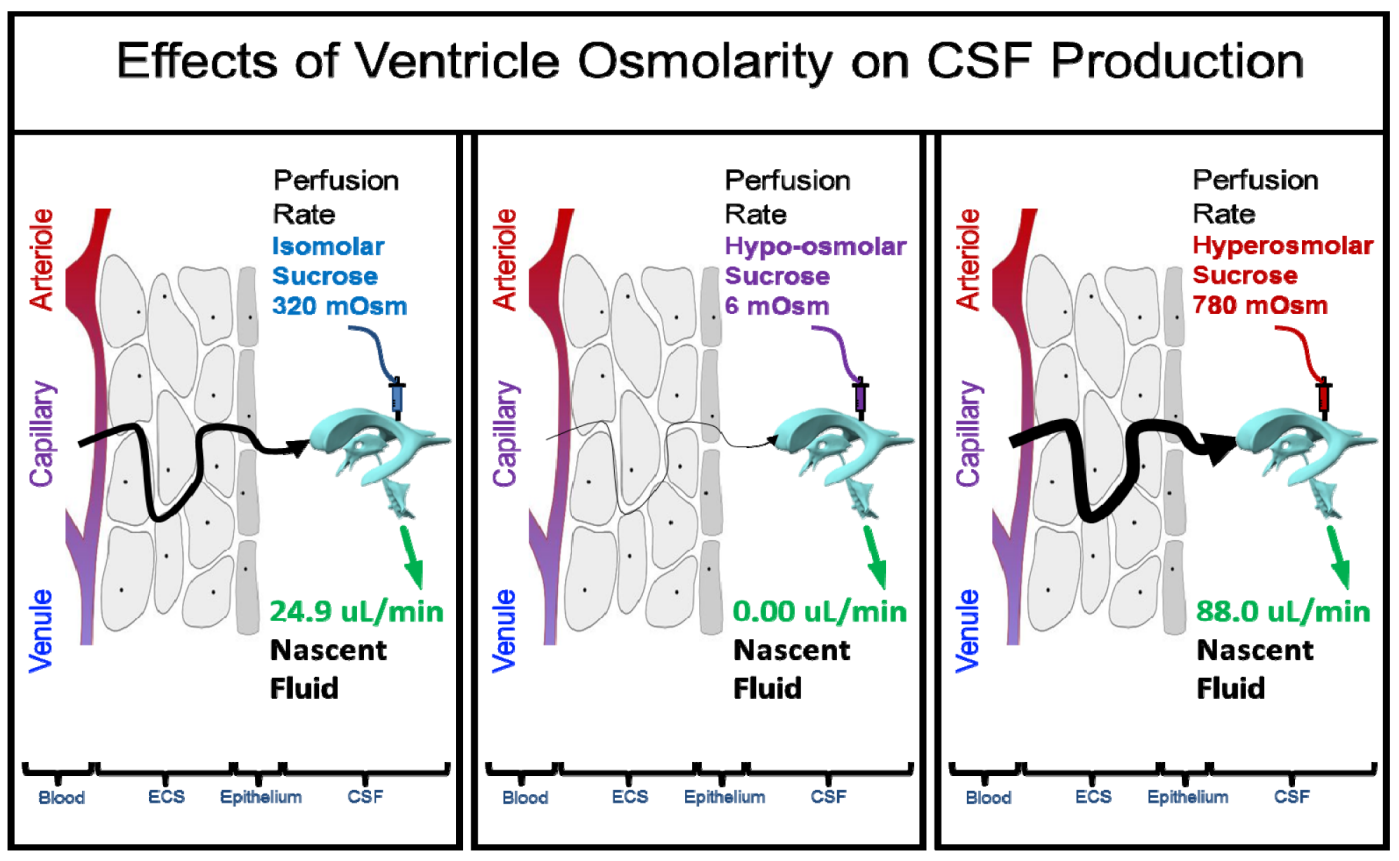
The effect of bolus injection of sucrose solution into the lateral ventricle during ventriculo-cisternal perfusion (VCP) in a feline model (25). Injection of hypo-osmolar solution was found to completely inhibit nascent fluid flow, while injection of hyperosmolar solution was found to increase the bulk flow of nascent fluid by up to 353%. The osmolarity of the ventricle was determined to alter bulk flow by 0.231 µL per mOsm as reported in the original experiment.

*E1. Ventricular osmolarity and the bulk flow of nascent fluid.* The results obtained by altering the osmolarity of the lateral ventricle via perfusion of a sucrose solution with osmolarities between 6-780 mOsm are illustrated by Figure 4 and Table 2. Control perfusion with a mock CSF of 320 mOsm produced a nascent fluid flow rate of 24.9 μL/min. A complete inhibition of bulk flow occurred with the injection of a 6 mOsm solution, while injection of a 780 mOsm solution increased bulk flow to 88.0 μL/min. Based on 29 experiments the osmolarity of the ventricle was determined to alter bulk flow by 0.231 µL per mOsm, as reported in the original experiment.

**Table 2:** Summary of the effect of serum and ventricle osmolarity on the bulk flow of nascent fluid during ventriculo-cisternal perfusion (VCP)

Experiment	Perfusion rate, µL/min	Injection, mOsm	Bulk flow, µL/min	Location
E1	77	6	0.0	Ventricle
		320	24.9	
		780	88.0	
E2	-	320	12.6	Ventricle
		950	24.8	
E3	77	290	49.9	Serum
		322	22.7	
		360	0.0	
E4	77	320	21.8	Serum
		60	54.9	
E5	12.6	320	12.6	Serum
		1110	0.9	

**Figure 7:**
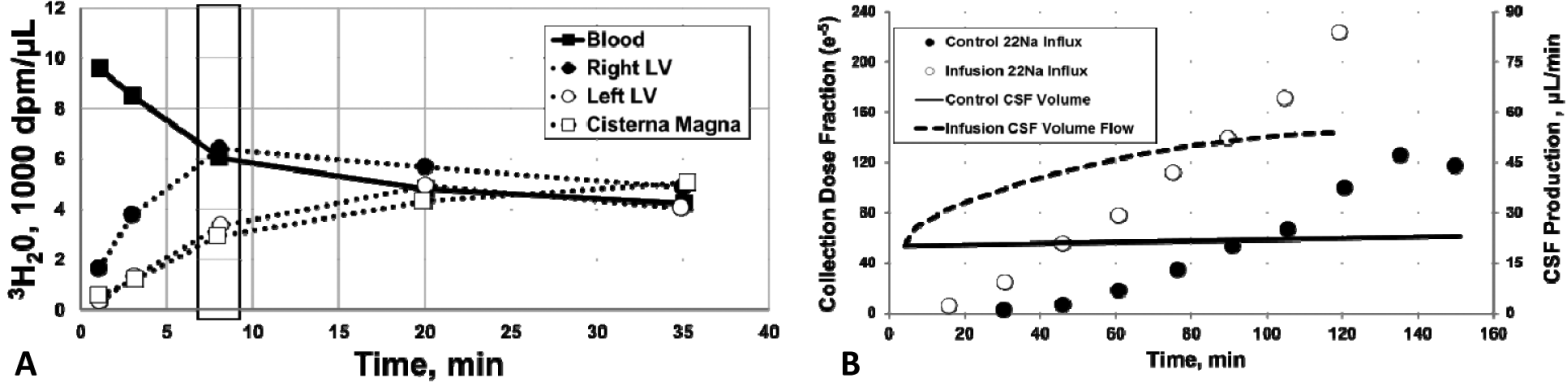
Experimental results showing cisterna magna water accumulation in response to osmotic pressure gradients. (**A)** Accumulation of radiolabeled water following a bolus injection of hyperosmolar solution into the right lateral ventricle (27), as described in experiment 2. Eight minutes post injection, as shown in the boxed region, the hyperosmolar right ventricle has received nearly twice the amount of radiolabeled water compared to the isotonic left ventricle. (**B**) The effect of a hypo-osmolar injection into the bloodstream on the bulk flow rate of nascent fluid and movement of radioactive solute from the extracellular space (ECS) into the cisterna magna during ventriculo-cisternal perfusion (VCP) (30) as described in experiment 4. The left y-axis represents the fraction of radioactivity of the collection fluid compared to the injection and the right y-axis represents the bulk flow of nascent fluid. Hypos-osmotic blood increases the rate of water and solute exchange between the ECS and doubles cerebrospinal fluid (CSF) production from 21.8 µL/min to 54.9 µL/min. LV – left ventricle.

*E2.Ventricular osmolarity and the fate of radiolabeled water.* Water transport in the brain was examined utilizing a combination of VCP and a bolus injection of radiolabeled water (^3^H_2_0) into the femoral vein of a canine model (27). An experimental control was established by a bolus injection of 320 mOsm sucrose solution isomolar to CSF, and radiolabeled tracer was measured in the cisterna magna over time. An injection of a 950 mOsm hyperosmolar sucrose solution into the right lateral ventricle increased the rate of radiolabeled water accumulation in the ventricle, effectively doubling the rate of CSF production from 12.6 µL/min to 24.80 µL/min, as shown in Table 2. This increase in CSF production led to a ventricular surface area enlargement by 2.1% shown in Table 3, as measured by planemetry. The raised osmolarity in the ventricle increased the rate of radiolabeled tracer accumulation when compared to the isomolar control, as shown in Figure 7A.

**Table 3:** Increase in ventricular volume due to chronic hyperosmolar solution injections into the lateral ventricle

Experiment	Author	Injection, mOsm	Duration, days	Surface increase, %	Volume increase, %
E2	Klarica (2013) (27)	2400	7	2.1	-
E6	Krishnamurthy (2009) (61)	307	12	-	0
		328	12	-	42
		337	12	-	83
		664	12	-	72
E6	Krishnamurthy (2012) (53)	337	15	-	125

**Figure 5:**
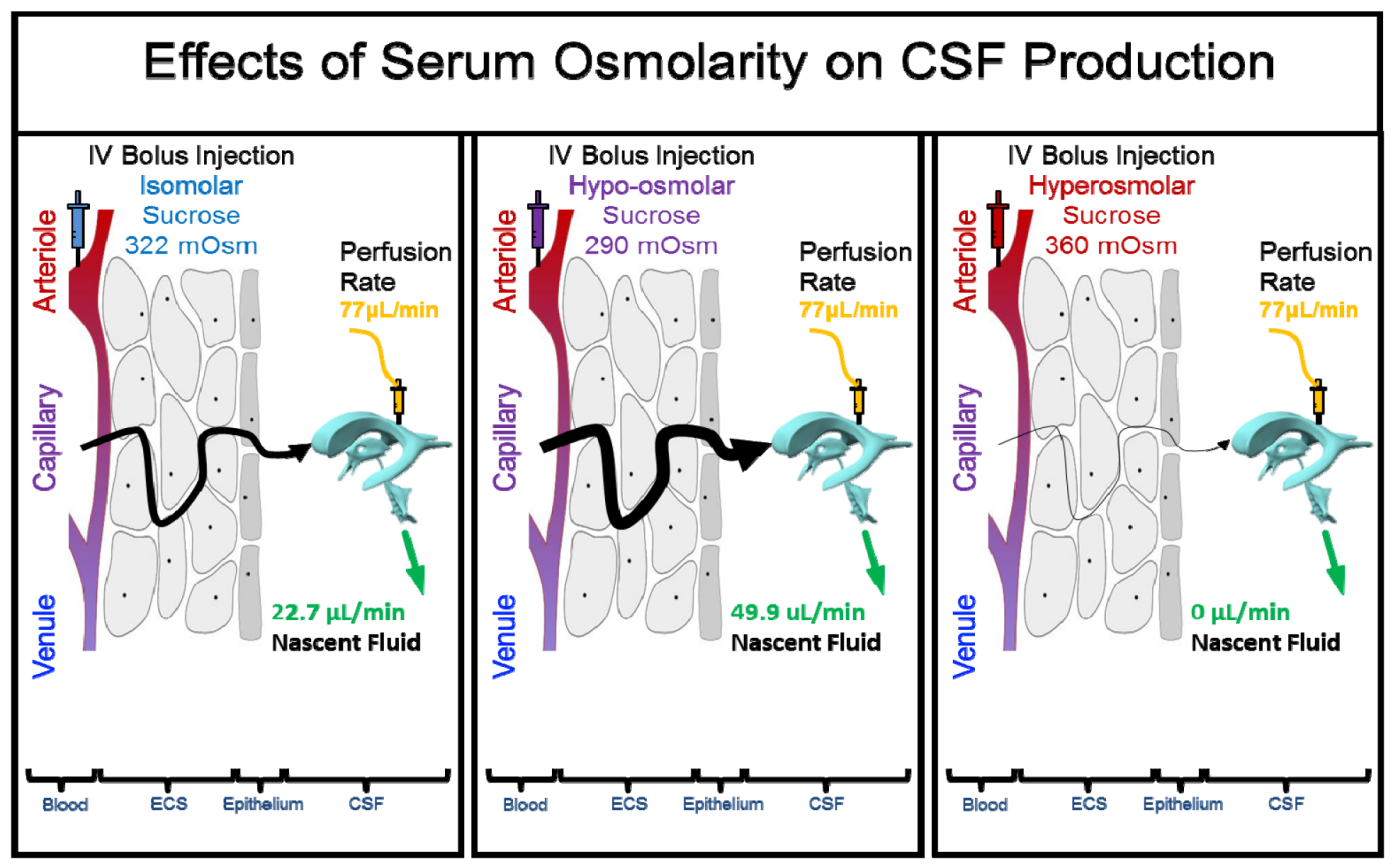
The effect of bolus injection of sucrose solution into the femoral artery during ventriculo-cisternal perfusion (VCP) in a feline model (26). Injection of hypo-osmolar solution was found to increase the bulk flow of nascent fluid up to 220%, while injection of hyperosmolar solution completely inhibited nascent fluid flow. The osmolarity of the serum was determined to alter bulk flow by 0.835 µL per Osm as reported in the original experiment.

*E3. Serum osmolarity and bulk flow of nascent fluid.* Experiment 3 examined the effect of serum osmolarity on the bulk flow of nascent fluid (26). The results obtained are shown in Figure 5 and Table 2. Bolus IV injection with the control solution of 322 mOsm yielded a bulk flow rate of 22.7 µL/min. The osmolarity of the serum was shown to completely inhibit the nascent fluid flow using a 360 mOsm injection, while flow was increased by 220% of the control when the osmolarity of the serum was decreased to 290 mOsm.

**Figure 6:**
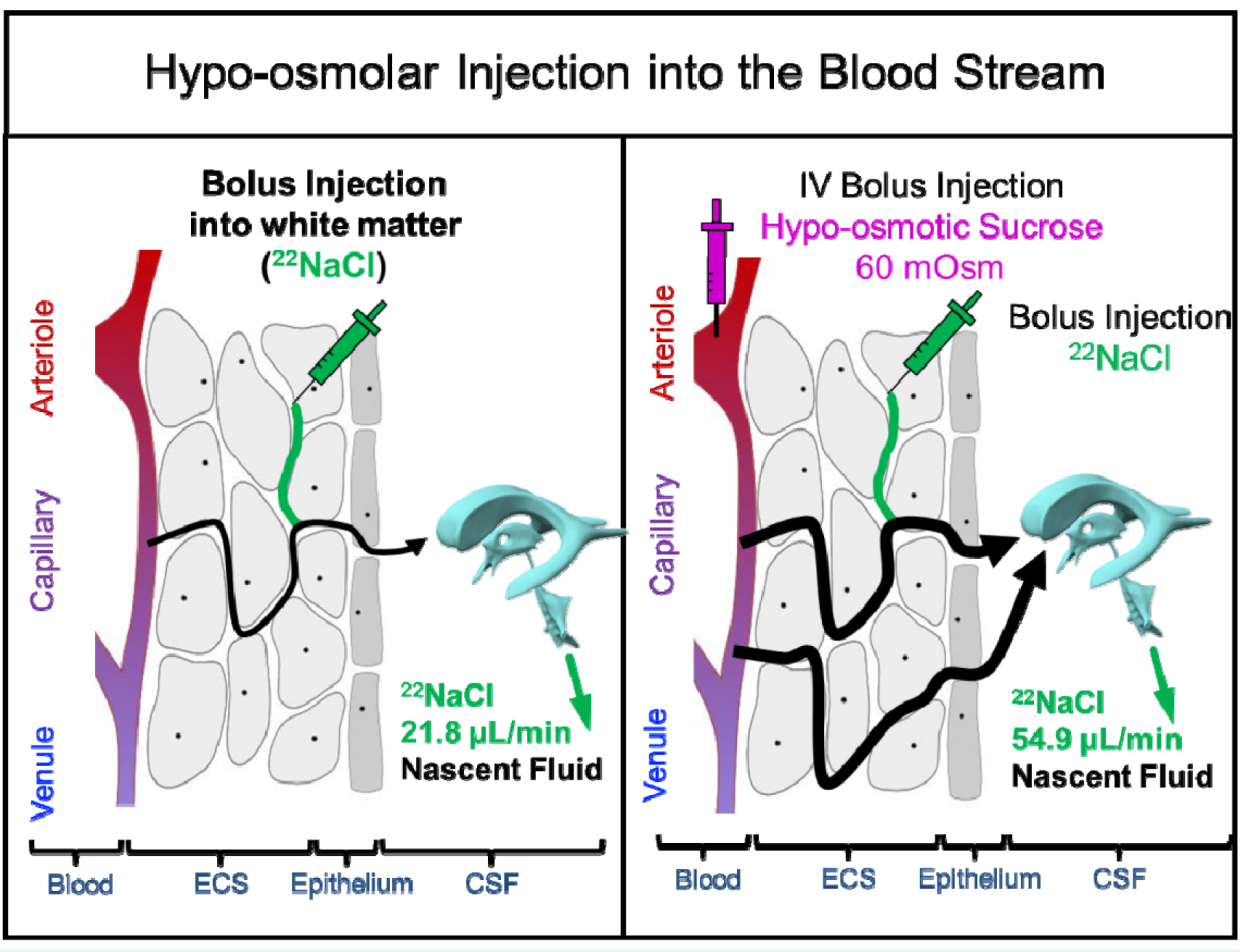
The effect of a hypo-osmolar injection into the bloodstream on the bulk flow rate of nascent fluid and movement of radioactive solute from the extracellular space (ECS) into the cisterna magna during ventriculo-cisternal perfusion (VCP) (30). Injection of hypo-osmolar solution was found to increase the flow rate of nascent fluid by a factor of 252%.

*E4. Serum osmolarity and the rate of solute transport from blood to CSF*. The results from experiment E4 performed by Wald et al (30) are demonstrated in Figure 6. Bolus injection of radiolabeled NaCl into the white matter during VCP with a perfusion of isomolar sucrose solution CSF led to an increase in radiolabeled NaCl concentration in the cisterna magna, but there was no change in nascent fluid production. Diluting the blood with a 6 mOsm injection led to an increase in nascent fluid production from 21.8 0 µL/min to 54.9 0 µL/min, as shown in Table 2.

Figure 7B shows the effects of this hypo-osmolar injection into the bloodstream on the production rate of CSF. This time-lapsed study of the movement of radioactive solute from the ECS into the cisterna magna shows that the collected fraction of the radiolabeled tracer increases when the osmolarity of the serum is decreased.

*E5. Increasing serum osmolarity and the bulk flow of nascent fluid.* The osmolarity of the serum had a significant effect on the bulk flow rate of nascent fluid into the ventricles. The injection of a 320 mOsm isomolar sucrose solution into the bloodstream led to a complete inhibition of nascent fluid flow. IV bolus injection of an 1110 mOsm mannitol solution decreased the bulk flow of nascent fluid by 93%, from 12.6 µL/min to 0.9 µL/min.

*E6. Effect of osmotic loading of the ventricle on ventricular volume.* The CSF osmolarity was shown to have a significant effect on ventricular volume, as measured by MRI, as shown in Table 3. By continuously injecting a 307 mOsm 40 kDa Dextran solution over 12 days, a ventricular volume growth of 42% was observed. A constant infusion of a 337 mOsm 10kDa Dextran solution increased the ventricular volume by nearly 83% over the course of 12 days, and increased the ventricular volume by up to 125% over 15 days (53). It was also shown that a continuous injection of a hyperosmolar fibroblast growth factor 2 solution of 664 mOsm over 12 days increased the volume of the ventricles by 72%.

**Figure 8:**
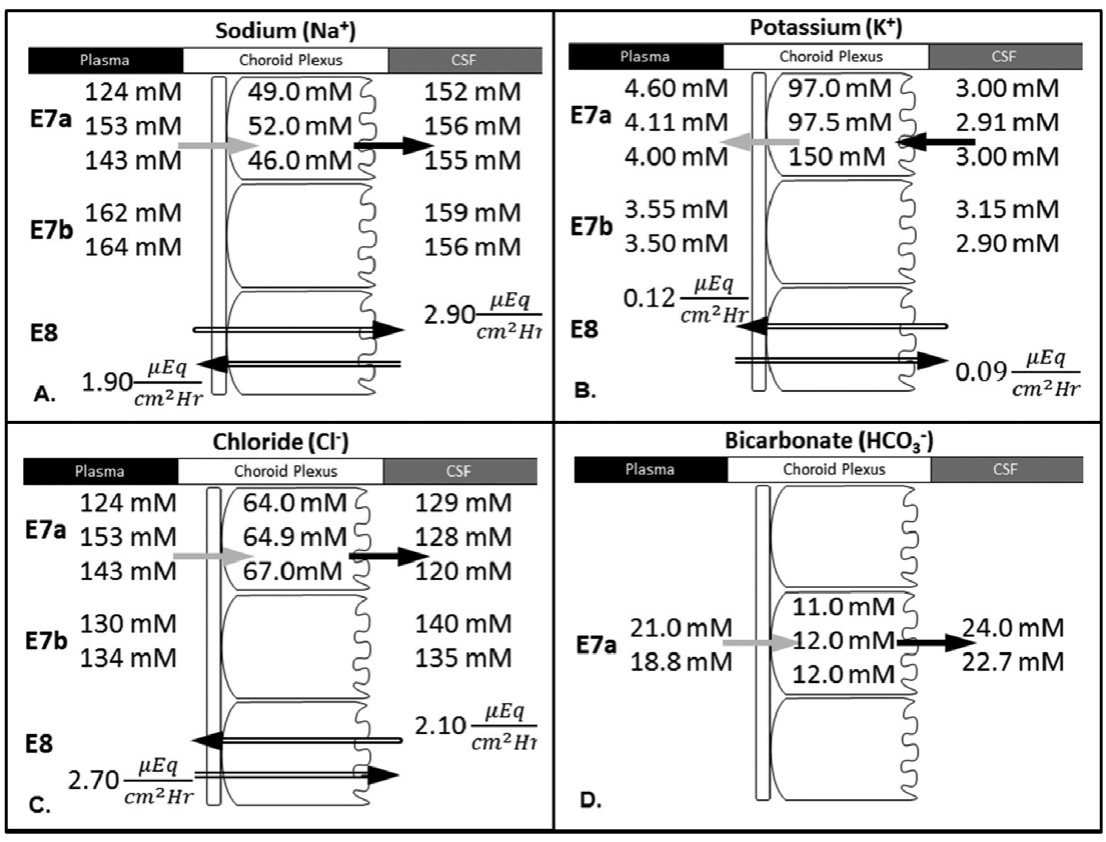
Data obtained from the measurements of Na^+^, K^+^, Cl^-^, and HCO_3_^-^ concentration in the serum, choroid plexus epithelium, and cerebrospinal fluid (CSF) and the flux of Na^+^, K^+^, and Cl^-^ between the CSF and plasma. Gray arrows indicate passive diffusion down a concentration gradient, black arrows indicate active transport, and striated arrows represent flux across the choroid plexus epithelium. The ionic concentration and flux of (**A**) sodium, (**B**) potassium, (**C**) chloride, and (**D**) bicarbonate are described. Experimental notation matches the previous sections.

*Measuring ionic concentration and flux in the blood, CSF, and choroid plexus (E7-E8).* The fluxes and concentrations of Na^+^, K^+^, Cl^-^, and HCO_3_^-^ in the serum, choroid plexus epithelium, and CSF (11-16) are illustrated in Figure 8. There was a net flux of Na^+^ and Cl^-^ across the choroid plexus epithelium into the CSF, while K^+^ flux was in the opposite direction from the CSF to the blood (8). The concentrations of Na^+^ and HCO_3_^-^ ions were higher in CSF than in either the plasma or the cells of the choroid plexus and chloride concentration in the plasma was higher than in the CSF. Intracellular concentration of K^+^ was much higher than in either the plasma or CSF (13,15,16).

## Discussion

This review of experimental evidence supports the exchange of water from either periventricular or choroid capillaries to the ventricles, known as the microvessel hypothesis. This theory is supported by carefully designed experiments that measure the production of nascent fluid by adjusting osmotic gradients between the CSF and vascular components of the brain. For the first time, we present a model that accounts for water exchange based on the following experimentally observed osmolarity differences. Measurements of bulk flow rates of nascent fluid during VCP and radiolabeled water injection experiments have demonstrated that both ventricles and blood exchange water at a rate influenced by osmotic gradients (25,27). Perfusion of sucrose solution into the lateral ventricle alters nascent fluid flow (25), raising the bulk flow of nascent fluid by 0.23l µL for each 1 mOsm. A bolus injection of sucrose solution into the femoral vein has been shown to alter nascent fluid production by 0.835 µL per Osm (26). These experiments indicate that the production of nascent fluid is more sensitive to osmolarity changes in the blood than in the CSF. It has also been shown with tracer experiments that transport of water and solute from the ECS to the cisterna magna is increased by diluting the plasma osmolarity (30).

Intracompartmental ion concentration experiments (11-16), coupled with the experiments by Wright et al (8), suggest that the choroid plexus is actively transporting ions between the blood and the CSF. The expression of Na^+^, K^+^, Cl, and HCO_3_^-^ transporters on both sides of the choroid plexus epithelium may explain the differences in ionic composition between the CSF and blood. This observation may elucidate the relatively constant ionic composition of CSF, which drives water filtration from periventricular capillaries into the ventricles

**Figure 9:**
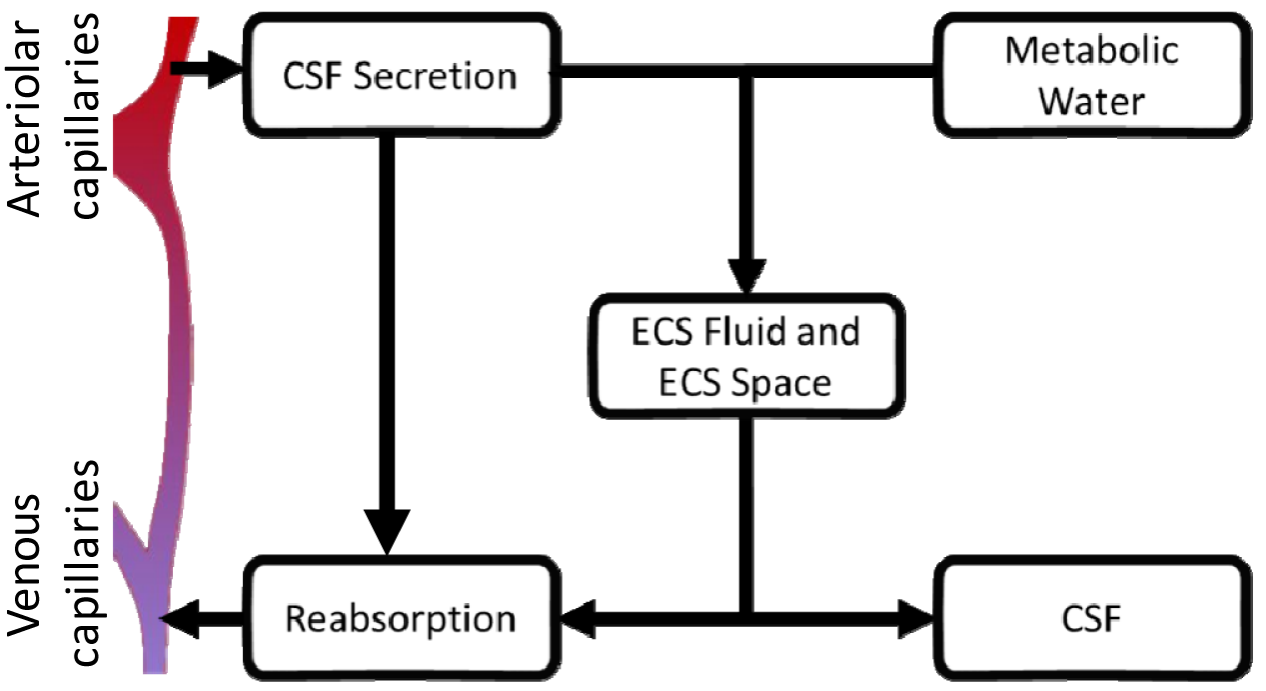
Extravascular water transport in the central nervous system (CNS). Water is filtered from arteriolar capillaries and moves into the extracellular space (ECS). Water is also generated by cellular metabolism. In the ECS, interstitial fluid can either be reabsorbed by the blood vessels or seep through ependymal layer of the ventricles to form nascent cerebrospinal fluid (CSF).

An additional source of water production in the ECS is the metabolism of glucose in the parenchyma described in Figure 9. Based on cerebral oxygen consumption measurements of 156 µmol/100g/min for the average human with a 1500 g brain, the total metabolic water produced (17,56,57) is 34 µL/min; roughly 49 mL per day. Compared to the total CSF production of 580 mL per day, this is a significant volumetric flux. This phenomenon must also be included in a phenomenologically accurate computational quantitative model of intracranial water dynamics.

**Figure 10:**
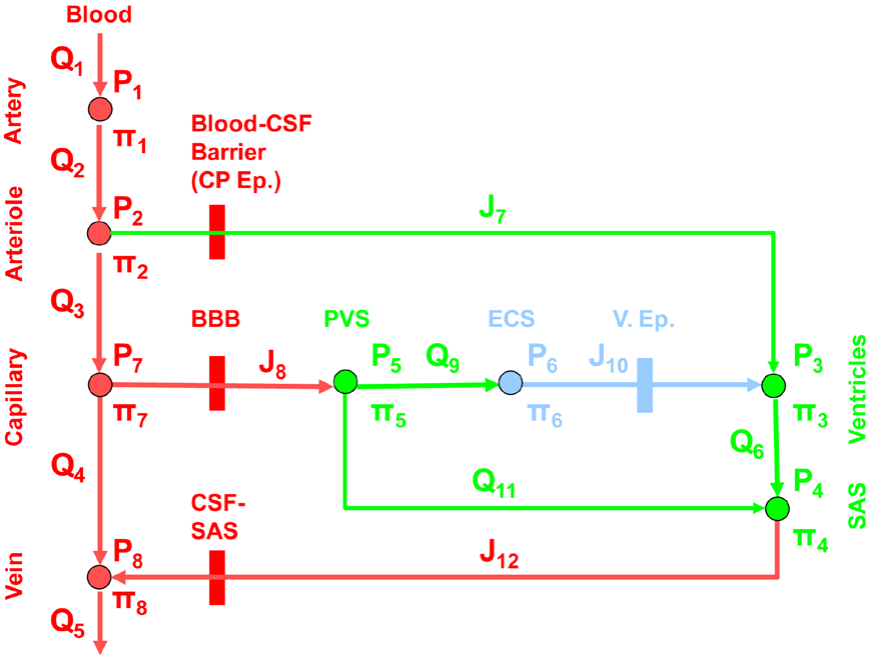
Network model for the flux of blood and water through the compartments of the brain simplified from the diagram in Figure 3. The vasculature is composed of an arterial, arteriolar, capillary, and venous compartment. Water and solute are filtered out of the arteriole blood through the choroid plexus ependymal (CP Ep.) blood- cerebrospinal fluid (CSF) barrier into the ventricles. Water and solute are also filtered from the capillaries through the blood brain barrier (BBB) into the perivascular space (PVS), where transport can occur between the extracellular space (ECS) and the subarachnoid space (SAS). Water moves from the ECS to the ventricles through the ventricular ependymal layer (V. Ep,). CSF moves from the ventricles through the aqueducts to the SAS. Water passes from the SAS through the arachnoid granulations at the CSF-SAS barrier into the veins, where it is drained. Water flux in the simplified network is either convection through the lumen of a blood vessel, Q1-6, 11, transmembrane flux, J7, 8, 10, 12, or transport through a porous medium, Q9. Hydrostatic pressure, P, and osmotic pressure, Π determine the water flux between each compartment.

A simplified network of 8 nodes and 12 arcs has been proposed to bring together these insights into a single model shown in Figure 10. This model describes the constant exchange of water between the blood, ventricles, and ECS. CSF production by the choroid plexus is supplemented by the filtration of water from cerebral capillaries, while reabsorption not only occurs via the AG but also locally into the blood, ventricle, or the ECS governed by Starling’s law. The following section describes a holistic mathematical model of water transport through the vascular, ECS, and CSF compartments as a function of both hydrostatic and osmotic pressure gradients. In this way, the model seeks to capture the relevant phenomena of both the classical and the microvessel models.

The simple network model shown in Figure 10 consists of vascular, ECS, CSF, and perivascular compartments. Each compartment is governed by a conservation balance and connecting arcs are assigned a phenomenological constitutive relationship. In the first section, the equations governing the mass conservation of bulk are discussed for water flowing through the vascular, ECS, CSF, and perivascular compartments. This transport is governed by osmolyte concentrations that are affected by the convection of the bulk volumetric flow rate of water. The second section describes the equations for computing the transport of different species between the vascular, ECS, CSF, and perivascular compartments that affect the osmotic pressure.

### Equations governing fluid exchange

By representing the vasculature and aqueducts as idealized cylinders with an equivalent hydrodynamic radius, the volumetric bulk flow is described by the Hagen Poiseuille equation shown in eq. 4, where the volumetric flow rate, Q, is dependent on the vessel radius, R; the vessel length, L; the hydraulic pressure drop across the vessel, ΔP; and the dynamic fluid viscosity, µ. Bulk flow described by eq. 4 occurs in the arteries, arterioles, capillaries, veins, and the aqueducts that connect the ventricles to the SAS. The main cause of the hydrostatic pressure gradient that drives volumetric blood flow computed by the Hagen Poiseuille equation is the arterio-venous pressure drop. This hydraulic pressure drop is smaller than the osmotic pressure gradient in certain scenarios. For example, the 5 mOsm osmolarity gradient between the plasma and the CSF results in a 20 mm Hg pressure difference. In terms of the osmotic counter pressure hypothesis, the hydrostatic pressure gradient driving fluid out of cerebral capillaries is roughly 10-30 mm Hg (17). The hydrostatic blood pressure drop from a 25 µm pre-capillary arteriole to a 25 µm post-capillary venule is also within this range (58). In comparison, dilution of the ECS by 1% would create a net osmotic pressure gradient of up to46 mm Hg (17) into the plasma.


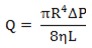
[4]

Water flux by Starling’s law occurs in the arterioles that feed the choroid plexus, capillaries, ECS, and ventricles. Water transfer across a membrane governed by Starling’s law is described by eq. 5, where the flux of water, J, is dependent on the hydraulic conductivity, L_p_, the reflection coefficient of the solute, σ, and the osmotic pressure gradient, Δπ. Both Q and J are responsible for the flow rate of water through the system; however J represents the filtration of water as a function of hydraulic and osmotic pressure gradients, whereas Q is dependent on hydraulic pressure gradients alone.


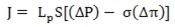
[5]

As the osmotic pressure gradients needed to construct the Starling’s law equations between intracranial compartments are difficult to determine, they are directly calculated from the concentration of osmolytes using the van’t Hoff equation. Osmolyte concentration is computed by solving a set of ordinary differential equations, which determine the convective transport of each osmolyte and conserve mass. As this solute convection is driven by the volumetric flux of water, which is dependent on Starling’s law, these phenomena are nonlinearly coupled, and must be solved simultaneously. These equations are described in the following sections.

*Mass conservation of water*. Figure 10 demonstrates the pathways for water in the brain. The equations used to describe the system include volumetric mass conservation equations for the flow of water, or molar conservation balances for the transport of proteins, ions, and glucose. The flow of water will be determined by applying a mass conservation balance at every node, as shown in eq. 6-13. The accumulation of volume in each compartment is represented by dV/dt, and the subscripts are as follows: Art is arteries, Arte is arterioles, Vent is ventricles, SAS is subarachnoid space, PVS is perivascular space, ECS is extracellular space, and Cap is capillaries. The bulk flux of water is given by Q, and J is the bulk flux out of the compartment driven by Starling’s law. The metabolic production of water in the ECS is given by G.


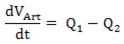
[6]


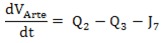
[7]


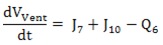
[8]


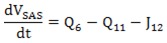
[9]


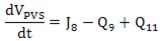
[10]


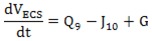
[11]


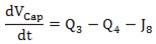
[12]


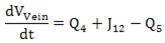
[13]

*Constitutive flux equations*. Constitutive flux equations are needed to determine the bulk flow of water between the compartments of the model where Q represents the bulk flux of water due to hydrostatic pressure differences and J is the flux driven by Starling forces. Volumetric bulk flow rate driven by hydrostatic differences is determined by the Hagen-Poiseuille law for eq. 14-19 and 24, where α is the equivalent hydrodynamic resistance. The bulk flow through the ECS is treated as a porous media and computed with Darcy’s law, where volumetric flow rate, Q, is determined by the cross-sectional area of the PVS-ECS interface, A_PVS-ECS_, the membrane permeability, k, and the hydraulic length through the porous media, L. All other fluxes are driven by Starling forces across membranes, shown by eq. 20, 21, 23, and 25. The concentration of each solute i contributes to the osmotic pressure gradient that drives Starling forces. The transport of solutes that provide major contributions to Starling forces are described in following sections. The hydrostatic pressure at the inlet and outlet are boundaries required by the degree of freedom analysis. As the reflection coefficient of the solute, σ, for each compartment may not be known a priori, these parameters may need to be fitted for using an optimization routine.


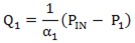
[14]


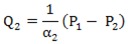
[15]


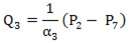
[16]


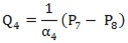
[17]


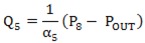
[18]


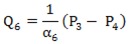
[19]


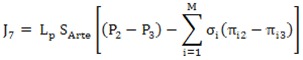
[20])


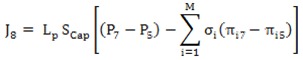
[21]


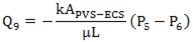
[22]


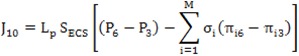
[23]


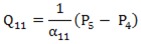
[24])


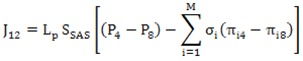
[25]

### Mass conservation of species

*Albumin transport*. The concentration of albumin in each compartment, C_alb_, is determined by constructing molar conservation balances for each compartment. Albumin convects freely through the vasculature and in between the ventricles and the SAS. Due to its large size, the protein will be limited in its transport through membranes by the albumin reflection coefficient, σ_Alb_, as shown in the following mass conservation equations eq. 26-33. The numerical values in the concentration subscripts are the same as for the osmotic pressure as shown in Figure 10. The concentration of albumin in the arterial blood is a boundary.


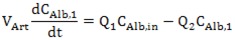
[26]


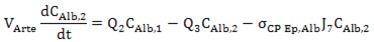
[27]


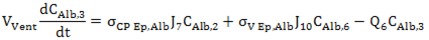
[28]


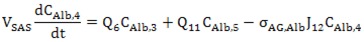
[29]


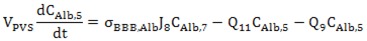
[30]


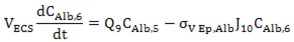
[31]


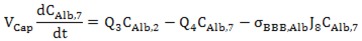
[32]


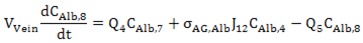
[33]

*Ion transport*. The transport of ions concentration in each compartment, C_ion_, is determined by constructing molar conservation balances as shown in eq. 34-41. For each ionic compound, this mass conservation must be solved, specifically the K^+^ and Na^+^ as they provide a significant contribution to the osmotic pressure in each compartment. Ions convect freely through the vasculature and in between the ventricles and the SAS. Though they are small enough to fit through the various junctions that connect these compartments, charge barriers exist as represented by the ionic reflection coefficient, σ_ion_. The concentration of ions in the arterial blood is a boundary.


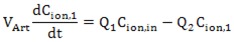
[34]


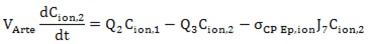
[35]


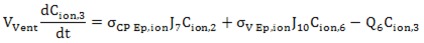
[36]


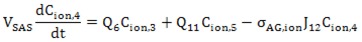
[37]


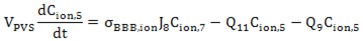
[38]


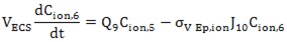
[39]


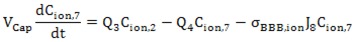
[40]


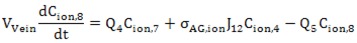
[41]

*Glucose transport*. Glucose transport throughout the intracranial system is computed by molar conservation balances as shown in eq. 42-49. Glucose concentration, C_glu_, provides a significant contribution to osmotic pressure, as well as to metabolic water production in the ECS determined by the first order destruction constant for glucose in the parenchyma, k_gluc,_ the concentration of glucose at the ECS node, C_gluc,6,_ and the volume of the ECS node, V_6_. Glucose transports freely in the vasculature and the aqueducts. However, its transport through membranes is mediated by active transport represented by the reflection coefficient, σ_Glu_. The concentration of glucose in the arterial blood is a boundary.


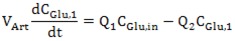
[42]


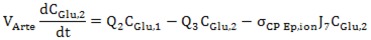
[43]


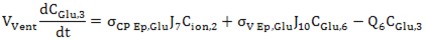
[44]


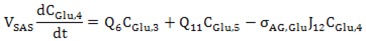
[45]


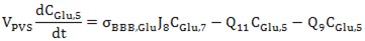
[46]


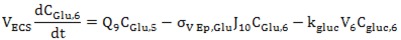
[47]


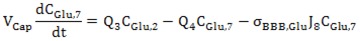
[48]


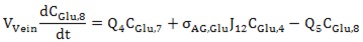
[49]

*Osmotic pressure determination.* The osmolarity of a solution can be calculated by equation 50, where the osmotic coefficient, φ, is related to the number of particles into which the molecule dissociates, n, and the molar concentration, C, for each solute in the solution, i. The osmotic coefficient accounts for the degree of non-ideal solute dissociation.


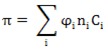
[50]

The determination of the osmotic pressure, π, is complicated by a number of factors that are derived from the discrepancy between ideal theoretical osmotic coefficients and measured osmotic coefficients (59,60). The osmotic coefficient depends on the temperature, amount fraction of solutes in solution, as well as the chemical potential of the pure solute and the solute in solution (59,60).

### Validation

The presented empirical data show the effect of osmolarity on the nascent fluid bulk flow per mOsm. Perfusion of sucrose solution into the lateral ventricle was shown to alter nascent fluid flow by 0.231 µL per mOsm (25), while bolus injection of sucrose solution into the femoral vein has been shown to alter nascent fluid production by 0.835 µL per Osm (26). The bulk flow of nascent fluid should be completely arrested by either a serum osmolarity of 360 mOsm or a ventricular osmolarity of 6 mOsm. An increase in the osmolarity of the ventricle should result in an increase in the volumetric bulk flow of water, while a continuous injection of hyperosmolar solution into the ventricular compartment should result in an accumulation of water in the ventricles, resulting in a volume increase (27,53,61).

Further validation of the model can be accomplished by computing the transport of radiolabeled water between the compartments in the brain. The time dependent concentration of ^3^H_2_O can be computed for ventricles, cisterna magna, and plasma. These data can be compared directly with the empirical data obtained in experiment E2 performed by Klarica et al (27), illustrated in Figure 7.

### Conclusion

Hydrocephalus, edema, and normal water drainage critically depend on osmotic pressure gradients between the compartments of the brain creating an abnormal accumulation of water. Hydrocephalus is an undesirable accumulation of water in the ventricles, while edema is associated with an accumulation of water in the ECS. It is possible to explain hydrocephalus by a pathologically high osmolarity in the ventricles drawing water from the blood or ECS, in fact, it has been shown (27,53,61) that a continuous injection of hyperosmolar solution alone can increase the volume of the ventricle up to 125% without blockage of the CSF pathway. The novel model presented here for the first time could provide a mathematical basis for this theory and elucidate the mechanisms of water exchange occurring in hydrocephalus and edema. The model can also explore the effects of increasing the osmolarity of the ECS on edema. Investigating the effect of Starling forces on water transport in the brain may yield clinically significant observations that lead to non-invasive treatment of conditions resulting from an imbalance in water movement in the brain.
